# Senecavirus A in Pigs, United States, 2015

**DOI:** 10.3201/eid2207.151591

**Published:** 2016-07

**Authors:** Ben M. Hause, Olivia Myers, Joshua Duff, Richard A. Hesse

**Affiliations:** Kansas State University, Manhattan, Kansas, USA (B.M. Hause, R.A. Hesse);; North Carolina State University, Raleigh, North Carolina, USA (O. Myers);; Maxwell Foods, Goldsboro, North Carolina, USA (J. Duff)

**Keywords:** Seneca valley virus, pigs, picornavirus, vesicular disease, Senecavirus A, United States, viruses

**To the Editor:** Senecavirus A (SVA) has been sporadically identified in pigs with idiopathic vesicular disease in the United States and Canada (*1*–*3*). Clinical symptoms observed include ruptured vesicles and erosions on the snout and lameness associated with broken vesicles along the coronary band. A recent report characterized SVA in pigs in Brazil with similar clinical symptoms in addition to a higher proportion of deaths than would be expected in pigs 1–4 days of age (*4*,*5*). Several outbreaks of this infection in pigs were reported in the summer of 2015 in the United States; the more severe clinical features resembled those seen in outbreaks in Brazil (*6*). Subsequent testing by PCR of 2,033 oral fluid samples from material submitted during 441 routine diagnostic testing procedures (from 25 states) identified 5 SVA-positive cases (1%) (*7*). Besides affecting animal health, SVA infection is notable because its clinical symptoms resemble those caused by foot-and-mouth disease and vesicular stomatitis viruses. When vesicular disease is observed in US swine, mandatory reporting and testing of animals for foreign animal diseases are required.

In June 2015, we collected 25 nasal and 25 rectal swab specimens from healthy pigs at 5 pig markets in North Carolina (250 total samples), representing pigs from 5 producers per market; the pigs were commingled for <12 hours. Primary markets 1 and 2 were slaughterhouses that purchased top quality pigs. Secondary market 3 was a slaughterhouse that purchased lower quality pigs (primarily underweight or herniated pigs). Market 4 was a broker that purchased pigs for culling and resold them for slaughter. Market 5 was a culled pig slaughterhouse. At markets 1–4, animals were ≈20 weeks of age; at market 5, animals were >10 weeks of age. 

We sampled the same sites a second time in August 2015. Again, we performed metagenomic sequencing on swab specimens pooled by producer (5 specimens per pool, 50 total pools per sampling) (*8*). Reads most similar to SVA were identified in numerous pools from samplings and at 4 different markets. Quantitative reverse transcription PCR (qRT-PCR) was performed at the Kansas State Veterinary Diagnostic Laboratory (Manhattan, KS, USA) on the original pooled samples and was positive for SVA (cycle threshold [C_t_] <37) for 26 (52%) pools from June and 18 (36%) pools from August. Sites 2 (n = 1 pool positive), 3 (n = 10), 4 (n = 5), and 5 (n = 10) had positive results in June, and sites 3 (n = 10), 4 (n = 1) and 5 (n = 7) had positive results in August. Both specimen types had an approximately equal number of positive results. We carried out virus isolation on swine testicle cells (positive samples from the second sampling), and 100% cytopathic effects were observed for 5 samples that tested positive for SVA by qRT-PCR with C_t_ values 16–21.

Templated assembly of the metagenomic sequencing reads with the SVA prototype strain SVV-001 genome (GenBank accession no. DQ641257) yielded near complete genomes from 5 pools (GenBank nos. KT827249–KT827253). The polyprotein-encoding region of the genomes showed >99% pairwise identity to each other and were most similar to sequences determined from recent outbreaks in Brazil (97%–98% nucleotide and >99% amino acid identity). Analysis of the P1 region of the genome found >99% nucleotide identity between 2015 US SVA sequences and 97% identity to SVA from Brazil. The contemporary US SVA sequences were more distantly related to SVA from an outbreak in Canada in 2011 (95% identity) and to historical US sequences (87%–92% identity). To investigate SVA phylogeny, we performed ClustalW (http://www.genome.jp/tools/clustalw/) alignment of P1 nucleotide sequences, followed by maximum-likelihood analysis using the best-fitting Kimura 2-parameter plus gamma distribution model of evolution. The 2015 US SVA sequences were most closely related to SVA sequences from Brazil; these sequences shared a common ancestor in Canada/11-55910-2011 (Figure).

**Figure:**
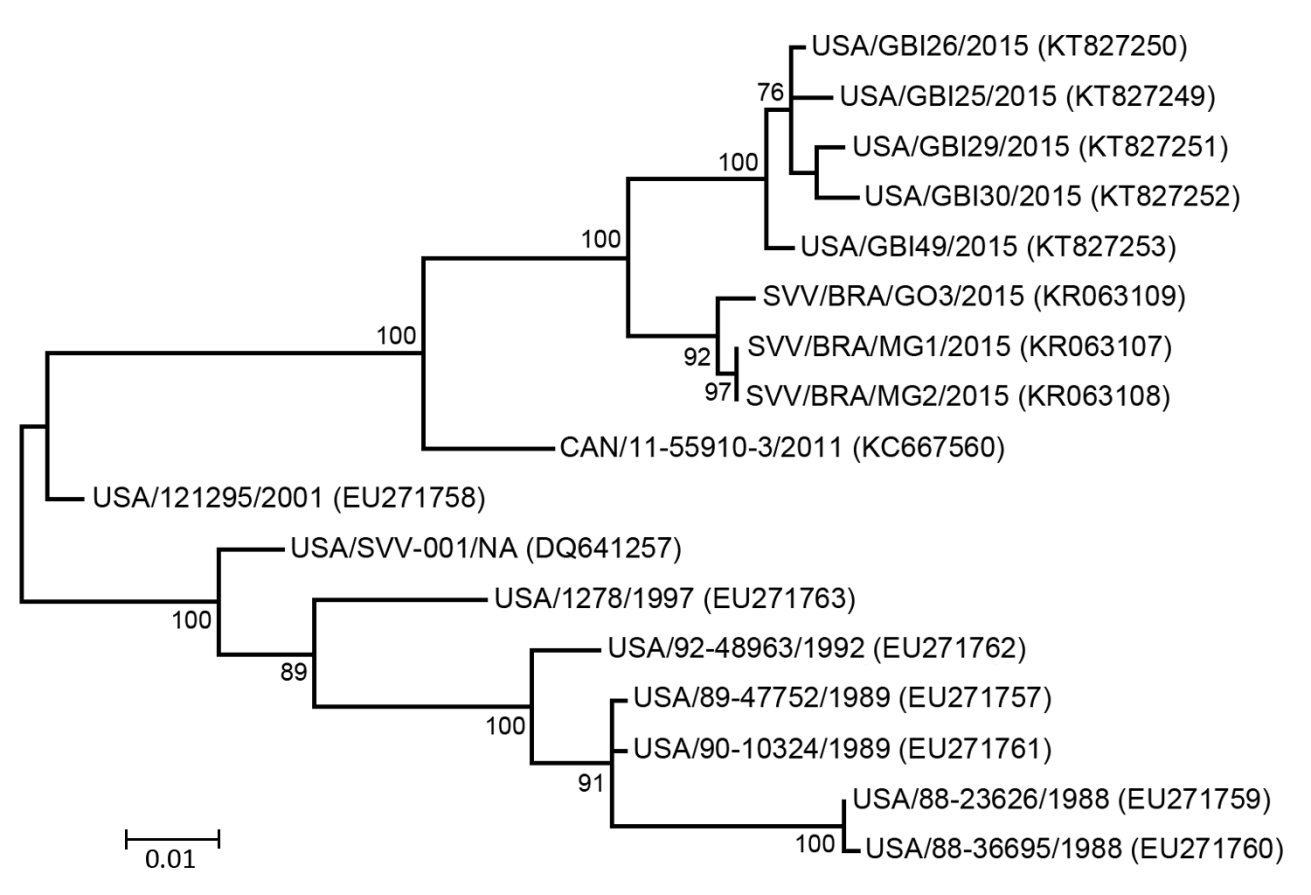
Phylogenetic tree of Senecavirus A P1 sequences. Maximum-likelihood analysis in combination with 1,000 bootstrap replicates as implemented in MEGA 6.06 (http://www.megasoftware.net) was used to derive the tree on the basis of nucleotide sequences. GenBank accession numbers are shown in parentheses. SVV in some isolate names indicates Seneca Valley virus, the original name for Senecavirus A. Scale bar indicates number of nucleotide changes per site.

Our results suggest that SVA commonly circulates in secondary and culled swine markets in North Carolina and that these strains are most similar to strains characterized in 2014–2015 in Brazil, which were associated with idiopathic vesicular disease and neonatal death. Little diagnostic testing is performed on culled animals, which may in part explain the discrepancy between 1% of oral fluids submitted for diagnostic testing being positive for SVA (*7*), compared with 72% of culled swine swab specimen pools in this study . The sole sample from primary markets that was positive for SVA by qRT-PCR had a C_t_ of 36.9, just below the negative cutoff of 37. 

Further research is needed to address possible correlation between SVA and health status of animals sold at lower value to cull markets. A notable distinction between contemporary SVA in the United States and Brazil, however, is that all the US samples originated from healthy animals that showed no clinical symptoms. Given the high genetic similarity between contemporary US SVA sequences and those from Brazil, additional cofactors likely affect clinical disease.
